# Dermoid Cyst of the Superolateral Preseptal Orbit in a Child: Ultrasound, MRI, and Histopathological Correlation

**DOI:** 10.7759/cureus.110013

**Published:** 2026-05-31

**Authors:** Zakaria Boulahcen, El Mahdi Choukri, Bouchra Dahmani, Siham Alaoui Rachidi

**Affiliations:** 1 Diagnostic and Interventional Radiology, Mohammed VI University Hospital, Tangier, MAR; 2 Radiology, Faculty of Medicine and Pharmacy, Abdelmalek Essaâdi University, Tangier, MAR

**Keywords:** histopathological correlation, magnetic resonance imaging, orbital dermoid cyst, orbital ultrasound, pediatric orbit, preseptal orbital lesion

## Abstract

Orbital dermoid cysts are benign congenital lesions that commonly arise along embryonic suture lines, particularly in the superolateral orbit. We report a case of a four-year-old boy presenting with a painless, progressively enlarging right superolateral orbital mass. Ultrasonography demonstrated a well-defined cystic lesion with heterogeneous hypoechoic content and fine internal echogenic foci, producing the characteristic “sack of marbles” appearance, with no significant internal vascularity on color Doppler imaging. Magnetic resonance imaging revealed a well-circumscribed superolateral preseptal orbital lesion with high signal intensity on T2-weighted and fluid-attenuated inversion recovery (FLAIR) images, diffusion restriction, and no appreciable internal post-contrast enhancement. The lesion was surgically excised, and histopathological examination confirmed the diagnosis of dermoid cyst. This case highlights the complementary roles of ultrasonography and magnetic resonance imaging in the diagnosis, anatomical localization, and pre-operative assessment of pediatric orbital dermoid cysts.

## Introduction

Orbital dermoid cysts are benign congenital lesions that result from sequestration of ectodermal tissue along embryonic lines of fusion or closure [1,2]. This embryologic origin explains their typical occurrence near craniofacial suture lines, particularly the frontozygomatic suture in the superolateral orbit [1-3]. Dermoid cysts are among the most common pediatric orbital lesions and represent a major proportion of childhood orbital cystic lesions requiring biopsy or surgical excision [3].

Clinically, superficial orbital dermoid cysts often present during childhood as painless, slowly enlarging, well-circumscribed periorbital or orbital masses [3-5]. In typical superficial lesions with clearly defined margins and no clinical signs of deeper extension, clinical assessment and ultrasonography may be sufficient for initial evaluation and surgical planning [3]. However, cross-sectional imaging is useful when lesion margins are poorly defined, deeper orbital extension is suspected, anatomical relationships are uncertain, atypical imaging features are present, or detailed pre-operative mapping is required [3,6,7].

Ultrasonography is useful as a first-line imaging modality in children because it is rapid, non-invasive, widely available, and avoids ionizing radiation. It can confirm the cystic nature of the lesion and assess internal and peripheral vascularity. Magnetic resonance imaging provides superior soft-tissue contrast and is particularly useful for defining preseptal versus retroseptal location, evaluating relationships with adjacent orbital structures, and assessing lesion content using conventional, diffusion-weighted, and post-contrast sequences [4,5]. Computed tomography may be useful when bone remodeling, erosion, calcification, or intraosseous extension is suspected, but its use in children should be balanced against radiation exposure [6,7].

This report describes a superolateral preseptal orbital dermoid cyst in a child, emphasizing the correlation between clinical presentation, ultrasonography, magnetic resonance imaging, surgical excision, and histopathological confirmation, while highlighting that MRI should be reserved for selected cases in which additional anatomical or diagnostic information is clinically useful.

## Case presentation

Clinical findings

A four-year-old boy was referred for evaluation of a right superolateral orbital mass that had shown continuous progressive enlargement over one year. There was no history of trauma, pain, fever, inflammatory signs, diplopia, tearing, or rapid episodic enlargement. Relevant birth history was not documented in the available medical record. There was no history of prior similar lesions and no family history of similar lesions.

Clinical examination revealed a firm, well-circumscribed, non-tender, mobile mass located in the superolateral aspect of the right orbit. The overlying skin was normal. Mild right upper eyelid ptosis was present. No limitation of ocular motility or exophthalmos was observed. Visual acuity was clinically preserved. Pupillary examination and fundoscopic findings were not documented in the available clinical record.

Imaging findings

Orbital ultrasonography demonstrated a well-defined cystic lesion in the right superolateral orbital region, measuring approximately 10×8×9 mm in three orthogonal planes. On B-mode ultrasound, the lesion showed heterogeneous hypoechoic content with fine internal echogenic foci, producing the characteristic “sack of marbles” appearance. This appearance refers to multiple small echogenic foci within cystic content, likely related to keratinous or sebaceous material. Color Doppler evaluation showed no significant internal vascularity, and no peripheral hypervascular rim was described. These findings supported a cystic, non-hypervascular lesion, although imaging alone could not establish the definitive histological diagnosis (Figures 1A-1C).

**Figure 1:**
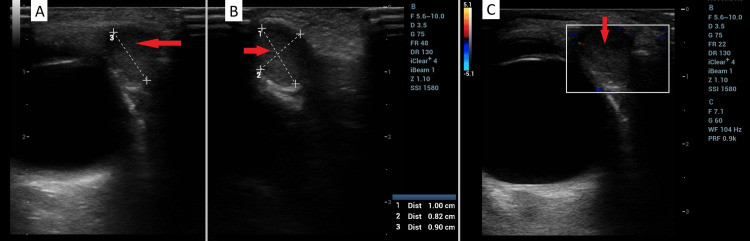
Orbital ultrasonography of the right superolateral orbital lesion. B-mode ultrasonography in axial (A) and sagittal (B) planes demonstrate a well-circumscribed cystic lesion in the superolateral aspect of the right orbit, with heterogeneous hypoechoic content and fine internal echogenic foci, producing the characteristic “sack of marbles” appearance. Color Doppler ultrasonography in the axial plane (C) demonstrates no significant internal vascularity. Red arrows indicate the cystic lesion.

Magnetic resonance imaging of the orbits demonstrated an oval, well-circumscribed lesion in the superolateral aspect of the right orbit, measuring approximately 11.4×8.3×10.2 mm in three orthogonal planes. The lesion was located in the preseptal compartment. This preseptal localization was important because it indicated that the lesion was superficial to the orbital septum, without retroseptal extension, which is relevant for surgical planning and prognosis. The lesion showed high signal intensity on T2-weighted images. It was in close anatomical contact with the lacrimal gland, superolateral eyelid margin, levator palpebrae superioris muscle, and lateral scleral surface of the globe, without definite imaging evidence of invasion of these structures. The globe, extraocular muscles, retroseptal fat, and optic nerve were preserved; the optic nerve was remote from the lesion. No retroseptal extension was identified (Figures 2A-2C).

**Figure 2:**
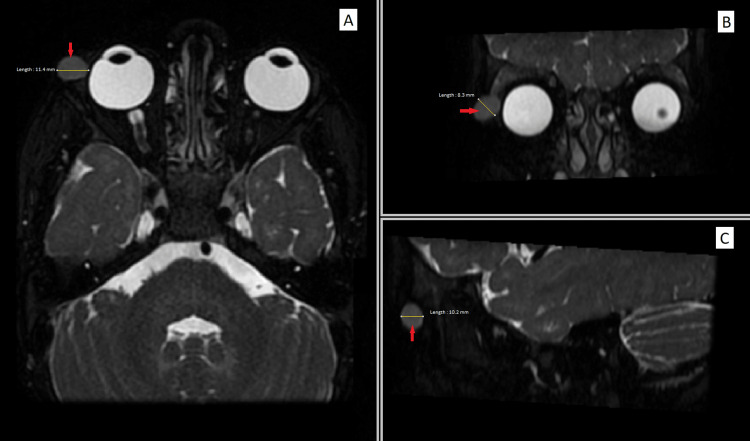
T2-weighted orbital MRI showing the anatomical location of the lesion. Axial (A), coronal (B), and sagittal (C) T2-weighted cube fat-suppressed images demonstrate a well-circumscribed cystic lesion in the superolateral preseptal compartment of the right orbit, measuring approximately 11.4×8.3×10.2 mm. There is no retroseptal extension. Red arrows indicate the lesion.

Additional brain and orbital MRI sequences demonstrated hyperintense signal on fluid-attenuated inversion recovery (FLAIR) imaging, no associated susceptibility artifact on enhanced susceptibility-weighted angiography (eSWAN) imaging, and diffusion restriction on diffusion-weighted imaging with corresponding low signal on the apparent diffusion coefficient map. These findings were compatible with a cystic lesion containing keratinous material, while recognizing that diffusion restriction is not specific and may also be observed in other keratin-containing lesions, particularly epidermoid cysts (Figures 3A-3D).

**Figure 3:**
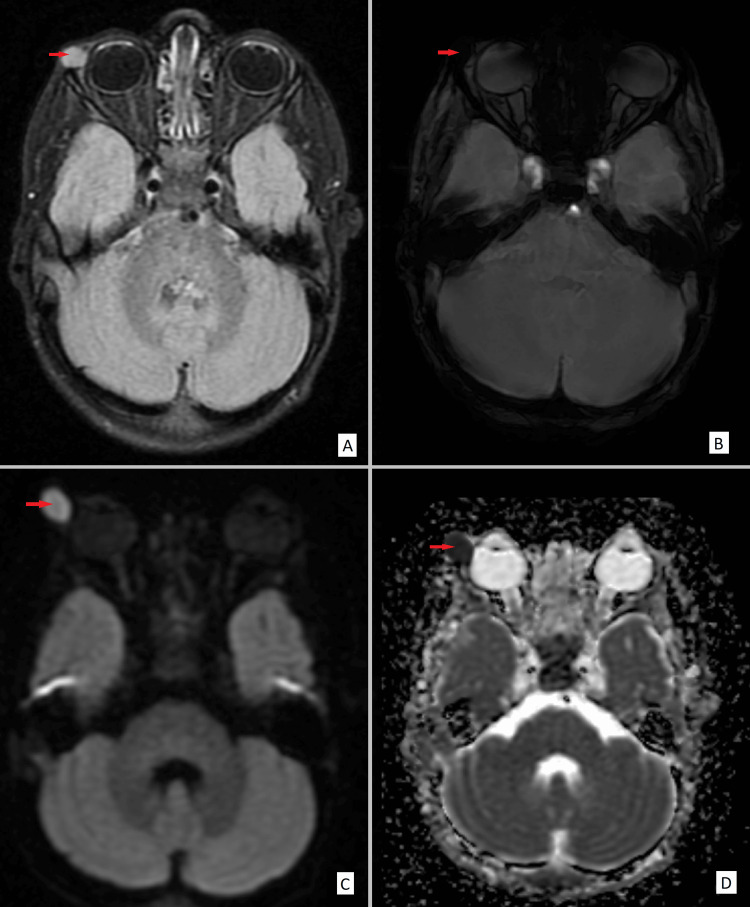
Brain and orbital MRI showing signal characteristics and diffusion assessment of the lesion. Axial FLAIR image (A) demonstrates hyperintense signal of the right superolateral orbital lesion. Axial eSWAN image (B) demonstrates no associated susceptibility artifact. Axial diffusion-weighted image (C) and corresponding apparent diffusion coefficient map (D) demonstrate diffusion restriction within the lesion. Red arrows indicate the cystic lesion. FLAIR: fluid-attenuated inversion recovery; eSWAN: enhanced susceptibility-weighted angiography

On T1-weighted imaging, the lesion was hypointense before contrast administration. Post-contrast fat-suppressed T1-weighted Dixon imaging showed no appreciable internal enhancement and no enhancing solid component. No definite enhancing rim was described. Based on the clinical presentation, continuous slow growth, superolateral preseptal orbital location, cystic ultrasonographic appearance, absence of significant vascularity, and MRI signal characteristics, an orbital dermoid cyst was the leading diagnosis. The main differential diagnoses considered at this stage included epidermoid cyst, dacryops, mucocele, lymphatic malformation, and abscess. An abscess was considered less likely because there were no clinical inflammatory signs, fever, pain, or a hypervascular rim. Lymphatic malformation was less likely because the lesion was well-circumscribed, unilocular, and non-hypervascular, without hemorrhagic or infiltrative features. Dacryops and mucocele were considered less likely given the lesion location, clinical context, and imaging morphology (Figures 4A, 4B).

**Figure 4:**
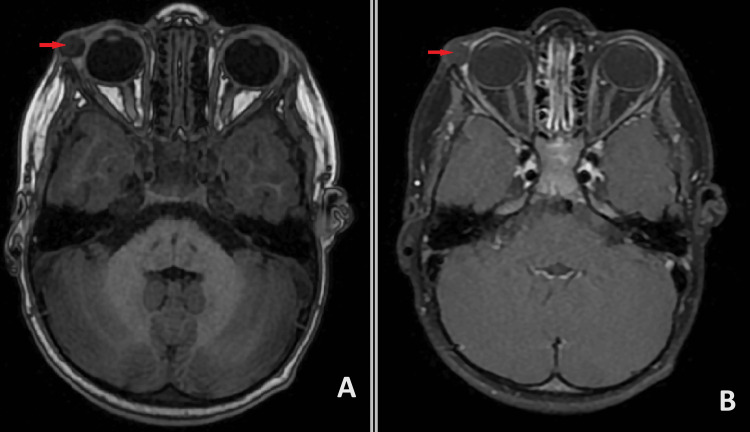
Pre- and post-contrast axial T1-weighted MRI of the orbital lesion. Axial non-fat-suppressed T1-weighted image before contrast administration (A) demonstrates a well-circumscribed hypointense lesion in the superolateral aspect of the right orbit. Axial fat-suppressed post-contrast T1-weighted Dixon image (B) demonstrates no appreciable internal enhancement and no enhancing solid component. Red arrows indicate the cystic lesion.

Management and histopathological findings

Based on the clinical and radiological findings, an orbital dermoid cyst was strongly suspected. The patient underwent surgical excision of the lesion. The exact surgical approach was not documented in the available operative record. No intra-operative rupture was reported, and no immediate post-operative complication was documented. Post-operative follow-up information regarding recurrence, cosmetic outcome, and ocular function was not available in the provided record.

Histopathological examination using hematoxylin and eosin staining demonstrated a cystic lesion containing keratinous material, lined by keratinizing stratified squamous epithelium with preservation of the granular layer and associated sebaceous adnexal structures. Hair follicles were not specifically documented. The presence of adnexal structures supported the diagnosis of a dermoid cyst and helped distinguish it from an epidermoid cyst, which is typically lined by squamous epithelium but lacks dermal adnexal structures. These findings confirmed the diagnosis of dermoid cyst (Figures 5A, 5B).

**Figure 5:**
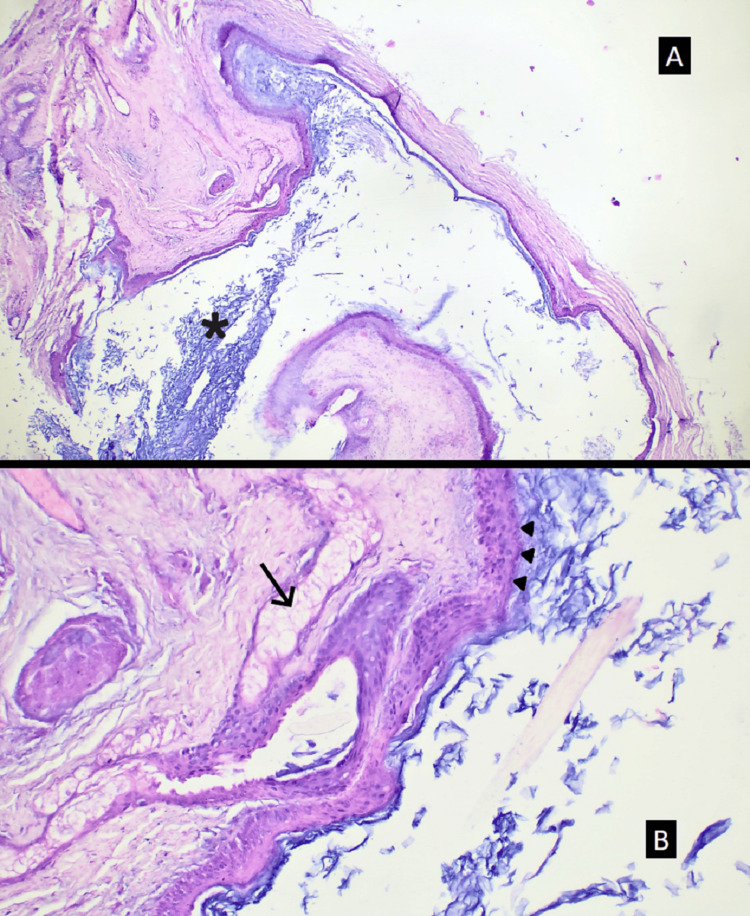
Histopathological confirmation of dermoid cyst. Hematoxylin and eosin-stained histological sections at low-power magnification ×40 (A) and high-power magnification ×200 (B) demonstrate a cystic lesion containing keratinous material, indicated by the black asterisk. The cyst is lined by keratinizing stratified squamous epithelium with preservation of the granular layer, indicated by black triangles, and associated sebaceous adnexal structures, indicated by the black arrow. These findings are consistent with a dermoid cyst.

## Discussion

Dermoid cysts are benign congenital lesions caused by sequestration of ectodermal tissue during embryologic fusion or closure [1-3]. In the orbit, this mechanism explains their frequent occurrence near craniofacial suture lines, particularly the frontozygomatic suture, which corresponds to the typical superolateral location observed in the present case [1-4]. Orbital dermoid cysts are among the most common pediatric orbital masses and represent an important proportion of childhood orbital cystic lesions encountered in surgical and biopsy series [3-5,8]. Although they most often occur in the orbit and periorbital region, dermoid cysts may also occur at other intracranial or craniofacial sites, including the anterior fontanelle, posterior fossa, parasellar region, and midline cranial locations [1,2].

The clinical presentation is usually that of a painless, slowly enlarging, well-circumscribed mass in early childhood [3-5]. In the present case, the lesion showed continuous progressive growth over one year, was mobile, non-tender, and located in the right superolateral orbit. The absence of pain, fever, inflammatory signs, diplopia, tearing, exophthalmos, and ocular motility limitation supported a benign superficial lesion. Mild ptosis was present, likely related to the local mass effect on the upper eyelid region. These clinical findings were consistent with a superficial orbital dermoid cyst, although imaging was required to define the lesion content, compartmental location, and relationship with adjacent structures.

Ultrasonography is often an appropriate first-line imaging modality for suspected superficial orbital dermoid cysts in children. It is rapid, non-invasive, does not require ionizing radiation, and can demonstrate whether a lesion is cystic or solid. In typical superficial lesions with well-defined clinical margins and no signs of deep extension, ultrasound combined with clinical assessment may be sufficient for initial evaluation and surgical planning [3-6]. In this case, ultrasonography demonstrated a well-circumscribed cystic lesion with heterogeneous hypoechoic content and fine internal echogenic foci, producing the “sack of marbles” appearance. This appearance likely reflects keratinous or sebaceous intracystic material. The absence of significant internal vascularity and the lack of a described peripheral hypervascular rim supported a non-hypervascular cystic lesion rather than an inflammatory or vascular process.

Magnetic resonance imaging is not required for every superficial orbital dermoid cyst. However, it is useful when there is uncertainty about lesion extent, preseptal versus retroseptal location, relationship with adjacent orbital structures, atypical clinical or ultrasound features, suspicion of deeper extension, or need for detailed pre-operative mapping [4-7]. In the present case, MRI confirmed that the lesion was located in the preseptal compartment, without retroseptal extension. This distinction is clinically important because preseptal lesions are generally more superficial and more surgically accessible, whereas retroseptal or deep orbital extension may increase operative complexity and the potential risk to orbital structures. MRI also demonstrated that the lesion was well-circumscribed and in close contact with adjacent superficial orbital structures without definite invasion.

The MRI signal characteristics were also helpful. The lesion showed high signal intensity on T2-weighted and FLAIR images, diffusion restriction with low apparent diffusion coefficient signal, and no appreciable internal post-contrast enhancement. These findings supported the presence of a cystic lesion containing keratinous or proteinaceous material. However, diffusion restriction should not be considered specific for dermoid cyst, because it may also be seen in epidermoid cysts and other keratin-containing lesions. Similarly, the absence of internal enhancement favors a benign cystic lesion but does not alone establish the diagnosis. Contrast-enhanced imaging in this case helped assess for an enhancing solid component, rim enhancement, or inflammatory features that could suggest alternative diagnoses such as abscess, lymphatic malformation with inflammation, or cystic neoplasm. Nevertheless, contrast-enhanced MRI should be used selectively and need not be performed routinely in every clinically typical superficial orbital dermoid cyst.

The main differential diagnoses of a pediatric orbital cystic lesion include epidermoid cyst, dacryops, mucocele, lymphatic malformation, and abscess [9]. The typical superolateral location, slow painless growth, cystic ultrasound appearance, absence of significant vascularity, preseptal location, lack of retroseptal extension, and absence of inflammatory signs favored a dermoid cyst in the present case. A concise comparison of imaging is provided in Table 1.

**Table 1:** Main differential diagnoses of pediatric orbital cystic lesions.

Diagnosis	Typical clues	Imaging features	Arguments in the present case
Dermoid cyst	Congenital lesion, slow painless growth, often superolateral near suture lines	Well-circumscribed cystic lesion; variable T1/T2 signal depending on lipid, keratin, or proteinaceous content; may restrict diffusion; usually no solid internal enhancement	Favored by superolateral preseptal location, slow growth, cystic appearance, no internal vascularity, and histopathological confirmation
Epidermoid cyst	Congenital keratin-containing lesion; may mimic dermoid cyst	Cystic lesion with marked diffusion restriction; lacks dermal adnexal structures histologically	Considered because of diffusion restriction, but histology showed sebaceous adnexal structures, supporting a dermoid cyst
Dacryops	Lacrimal ductal cyst, usually related to lacrimal gland region	Thin-walled cystic lesion near lacrimal gland; usually simple fluid signal	Less likely because the lesion had heterogeneous internal content and histology confirmed dermoid cyst
Mucocele	Often related to paranasal sinus disease or bone remodeling	Expansile cystic lesion, often adjacent to sinus; may remodel bone	Less likely because of superficial preseptal superolateral location and lack of sinus-centered features
Lymphatic malformation	May enlarge episodically, sometimes with hemorrhage	Multiloculated infiltrative lesion; fluid-fluid levels may be present; variable enhancement	Less likely because the lesion was well-circumscribed, unilocular, non-hypervascular, and not episodically enlarging
Abscess	Pain, fever, inflammatory signs, acute presentation	Rim-enhancing collection with surrounding inflammatory change; may show restricted diffusion	Less likely because there was no fever, pain, inflammatory signs, or definite enhancing rim

Histopathology remains the definitive diagnostic method. Dermoid cysts are lined by keratinizing stratified squamous epithelium and contain dermal adnexal structures, such as sebaceous glands or hair follicles. Epidermoid cysts may also be lined by squamous epithelium and contain keratinous material, but they lack dermal adnexal structures. In the present case, the presence of sebaceous adnexal structures confirmed a dermoid cyst and helped distinguish it from an epidermoid cyst.

Complete surgical excision is the usual treatment for symptomatic, enlarging, cosmetically significant, or diagnostically uncertain orbital dermoid cysts [3-5]. Careful excision is important because rupture may release keratinous or sebaceous material and trigger a granulomatous inflammatory reaction. Recurrence is mainly associated with incomplete excision or residual cyst wall. Observation may be appropriate in selected small, asymptomatic, clinically typical superficial lesions, especially when the lesion is stable and not causing functional or cosmetic concerns [10]. Therefore, management should be individualized according to lesion size, growth, symptoms, cosmetic effect, imaging features, and surgical accessibility.

In this case, surgical excision was performed, and no intra-operative rupture or immediate post-operative complication was reported. Histopathological findings were concordant with the radiological diagnosis. However, post-operative follow-up data regarding recurrence, cosmetic outcome, and long-term ocular function were not available in the provided record. This represents a limitation of the report. Another limitation is the absence of clinical photographs, which were not available for inclusion. Despite these limitations, the case illustrates the complementary value of clinical examination, ultrasonography, MRI, surgical excision, and histopathological confirmation in a pediatric superolateral preseptal orbital dermoid cyst, while emphasizing that MRI should be reserved for selected cases in which additional anatomical or diagnostic information is clinically useful.

## Conclusions

A superolateral preseptal orbital dermoid cyst should be considered in children presenting with a slowly enlarging, painless orbital or periorbital mass. Ultrasonography is a useful first-line imaging modality for confirming the cystic nature of the lesion and assessing vascularity, particularly in clinically typical superficial lesions. Magnetic resonance imaging is not required for every superficial orbital dermoid cyst, but it can be valuable when the extent of the lesion, preseptal versus retroseptal localization, atypical features, or relationships with adjacent orbital structures need to be clarified for diagnosis or surgical planning. Surgical excision allows definitive histopathological confirmation, and careful removal is important to reduce the risk of rupture-related inflammation or recurrence.

## References

[1] Kudo K, Tsutsumi S, Suga Y, Okura H, Abe Y, Yasumoto Y, Ito M (2008). Orbital dermoid cyst with intratumoral inflammatory hemorrhage: case report. Neurol Med Chir (Tokyo).

[2] Ahmed RA, Eltanamly RM (2014). Orbital epidermoid cysts: a diagnosis to consider. J Ophthalmol.

[3] Pushker N, Meel R, Kumar A, Kashyap S, Sen S, Bajaj MS (2020). Orbital and periorbital dermoid/epidermoid cyst: a series of 280 cases and a brief review. Can J Ophthalmol.

[4] Kim DH, Prat DL, Tadros S, Katowitz WR (2022). Orbital and periorbital dermoid cysts: a retrospective analysis of 270 lesions. Orbit.

[5] Cavazza S, Laffi GL, Lodi L, Gasparrini E, Tassinari G (2011). Orbital dermoid cyst of childhood: clinical pathologic findings, classification and management. Int Ophthalmol.

[6] Chawda SJ, Moseley IF (1999). Computed tomography of orbital dermoids: a 20-year review. Clin Radiol.

[7] Nugent RA, Lapointe JS, Rootman J, Robertson WD, Graeb DA (1987). Orbital dermoids: features on CT. Radiology.

[8] Bansal R, Honavar SG, Talloju SS, Mulay K (2022). Orbital dermoid cyst. Indian J Ophthalmol.

[9] Tooley AA, Tailor P, Tran AQ, Garrity JA, Eckel L, Link MJ (2022). Differentiating intradiploic orbital dermoid and epidermoid cysts utilizing clinical features and machine learning. Indian J Ophthalmol.

[10] Shields JA, Shields CL (2004). Orbital cysts of childhood - classification, clinical features, and management. Surv Ophthalmol.

